# Cumulative Anticholinergic Burden and Risk of Delirium Among Older Adults with Alzheimer’s Disease

**DOI:** 10.3390/pharmacy14040089

**Published:** 2026-06-23

**Authors:** Ashna Talwar, Jeffrey Sherer, Susan Abughosh, Satabdi Chatterjee, Rajender R. Aparasu

**Affiliations:** 1 Department of Pharmaceutical Health Outcomes and Policy, College of Pharmacy, University of Houston, Houston, TX 77204-5047, USA; ashnatalwar1@gmail.com (A.T.); smabugho@central.uh.edu (S.A.); schatte3@central.uh.edu (S.C.); 2 Department of Pharmacy Practice and Translational Research, College of Pharmacy, University of Houston, Houston, TX 77204-5047, USA; jtsherer@central.uh.edu

**Keywords:** Alzheimer’s disease, delirium, cumulative anticholinergic burden, cholinesterase inhibitors, generalized boosted models

## Abstract

Delirium is a transient neuropsychiatric condition that is a severe and prevalent condition affecting 2.6 million older adults each year. Alzheimer’s disease (AD) and anticholinergic medication use are risk factors for delirium. This study evaluated the association between cumulative anticholinergic burden (CAB) and risk of delirium among older adults with AD initiating cholinesterase inhibitors (ChEIs). This retrospective cohort study used 2013–2017 Medicare claims data, and included adults 65 years and older with AD who initiated any of the ChEIs (donepezil, rivastigmine, and galantamine) after a 12-month washout period. CAB, as the primary exposure, was measured on the index date and calculated as the monthly total standardized daily dose of anticholinergic medications. A multivariable Cox proportional hazards regression model with inverse probability of treatment weighting (IPTW) generated using generalized boosted models was used to evaluate the risk of delirium associated with the CAB. This study identified 143,320 older adults with AD who initiated ChEIs. Most patients were in the low/no burden (62.73%) group, followed by high burden (21.12%) and moderate burden (16.14%). Overall, delirium diagnosis was observed in 19.11% of the cohort. The Cox regression model with IPTW found that moderate (aHR, 1.56; 95% CI, 1.52–1.61; *p* < 0.0001) and high CAB (aHR, 1.45; 95% CI, 1.42–1.49; *p* < 0.0001) were associated with an increased risk of delirium compared to low/no burden. Among older adults with AD initiating ChEIs, moderate and high CAB were associated with an increased risk of delirium compared with low/no CAB. These findings highlight the need to carefully reduce the CAB, especially dose and duration, along with utilizing anticholinergic alternatives in older adults with AD.

## 1. Introduction

Delirium is a transient neuropsychiatric condition characterized by inattention and fluctuations in cognition and consciousness. Though acute and abrupt, it is a severe and prevalent condition affecting 2.6 million older adults each year [1]. Delirium is associated with accelerated cognitive decline, increased risk of medical complications, institutionalization, functional decline, and longer hospitalizations, resulting in an annual cost of $164 billion in the US [2]. Rates of morbidity and mortality are high among patients with delirium, with 35–40% of hospitalized patients with delirium dying within one year [3]. Although the pathogenesis of delirium is not fully understood, it is postulated that multiple neurotransmitter abnormalities, especially cholinergic deficiency, are the precipitating factors [4]. Older adults (65 years or older) with an underlying neurocognitive disorder such as dementia/Alzheimer’s disease (AD) are at high risk of developing delirium due to an impairment in cholinergic transmission [5]. A meta-analysis found that delirium increased the risk of mortality by 75% in patients with dementia [6].

Medications with anticholinergic properties act by inhibiting the action of acetylcholine at cholinergic receptors, thereby adding to the cholinergic deficit in AD. Older adults with AD, when exposed to anticholinergic medications, exhibited worse cognitive scores than those without exposure [7]. Anticholinergic medications are an independent risk factor for delirium and, hence, are associated with worsening of cognition, functional decline, and increased mortality [8]. Many validated tools have been developed to quantify the anticholinergic burden. The SHELTER study concluded that anticholinergic burden as measured by the Anticholinergic Cognitive Burden (ACB) scale was associated with an increased risk of delirium in nursing home residents with dementia [9]. This study found that among patients with dementia, delirium prevalence increased with increasing anticholinergic burden according to the ACB scale, from 20% (with no anticholinergic burden) to 25% (with moderate burden) and 27% delirium (with strong burden scores) [9].

Several studies suggested a significant association between anticholinergic use and delirium in older adults with cognitive impairment/dementia/AD [10,11,12,13,14,15,16]. A study among Turkish patients with delirium superimposed on dementia found that the increase in sedative and anticholinergic medication burden increased the hazards of delirium by four-fold (Hazards ratio: 4.09) [15]. Another study found a 52% increased risk of delirium associated with high anticholinergic burden in older Korean adults with mild-to-moderate AD [14]. However, the research by Campbell and colleagues did not find any association between anticholinergic use and delirium [13]. Cholinesterase inhibitors (ChEIs) such as donepezil, rivastigmine, and galantamine, the mainstay pharmacotherapy for mild to moderate AD, inhibit acetylcholinesterase and thereby enhance cholinergic transmission [17]. The co-administration of ChEIs and anticholinergic medications can lead to drug–drug interaction, nullifying the treatment benefit of ChEIs and worsening AD due to the therapeutically opposing mechanisms of action [18,19]. However, no studies have evaluated the risk of delirium using cumulative anticholinergic burden (CAB) in patients concomitantly prescribed with ChEIs. The CAB captures the collective effect of multiple medications having anticholinergic properties, which can have adverse effects on the patient. This study aims to evaluate the risk of delirium associated with CAB after initiating ChEIs among older adults with AD.

## 2. Methods

### 2.1. Data Source and Study Design

This study used 2013–2017 Medicare claims data including inpatient, outpatient, prescription drug, demographic, and enrollment files [20]. This study was approved under the exempt category by the University of Houston Committee for the Protection of Human Subjects. We conducted a retrospective cohort study of older adults with AD to examine the association between CAB and risk of delirium after ChEI initiation. Eligible beneficiaries were required to have uninterrupted fee-for-service Medicare coverage, including Parts A, B, and D, with no HMO enrollment during the 12-month baseline period before their first observed ChEI fill, which served as the index date. Beneficiaries were eligible for inclusion if they were at least 65 years old, had evidence of AD based on diagnosis codes (International Classification of Diseases Ninth Revision (ICD) 9-CM 331.0 [21], ICD-10-CM G20, and F00 [22]), and maintained continuous Medicare Parts A, B, and D enrollment throughout the 12-month baseline period preceding ChEI initiation. We excluded beneficiaries with evidence of ChEI use during baseline to restrict the cohort to incident ChEI users. We also excluded patients with baseline memantine use, either as monotherapy or combination therapy, to reduce the inclusion of individuals with more advanced AD. Patients with delirium during baseline were excluded to focus on incident delirium during follow-up. Further, delirium events within one month after the index date were excluded so that we could account for the monthly exposure before the outcome occurred. Patients were followed for one year from the index date until the first occurrence of the event of interest (delirium), disenrollment from the health plan, change in the level of ACH burden, death, discontinuation of ChEI, or end of the one-year follow-up period. Reporting was guided by the Strengthening the Reporting of Observational Studies in Epidemiology (STROBE) recommendations for observational cohort studies [23].

### 2.2. Exposure and Outcome Definitions

The primary exposure was CAB during the first 30 days after the index date. CAB was calculated using a previously published approach described in detail in our earlier work [24,25]. This method combines medication-specific anticholinergic potency with patient-level dose exposure derived from prescription claims [26,27].

Anticholinergic potency was assigned using the ACB scale, which scores medications from 0 to 3, with higher scores indicating greater anticholinergic activity [28]. Anticholinergic medications were identified using National Drug Codes from Medicare Part D prescription claims [29,30,31]. The medications included in the CAB calculation are listed in Supplementary Table S1.

Patient-specific exposure was derived using prescription fill date, strength, quantity dispensed, and days supplied, as described in Supplementary Table S2 [32]. For each anticholinergic medication, the standardized daily dose was weighted by the medication’s corresponding ACB score to generate drug-specific standardized daily anticholinergic exposure. Monthly drug-specific exposure values were then summed to calculate the total standardized daily anticholinergic exposure sum, referred to as SumSDACE.

CAB during the first month after ChEI initiation was used as the exposure of interest. During follow-up, patients were censored in the month after a change in SumSDACE category. Because established monthly SumSDACE thresholds for older adults with AD are not available, burden categories were defined based on the distribution of values in the study cohort. SumSDACE was classified as no burden, low burden, moderate burden, or high burden using the following thresholds: no burden: SumSDACE = 0; low burden: SumSDACE ≤ 10; moderate burden: SumSDACE 10.1–40; and high burden: SumSDACE > 40. For analysis, the no- and low-burden groups were combined and used as the reference category.

The outcome, delirium, was identified using ICD-9-CM [33] and ICD-10-CM [34] diagnosis codes selected from the validated literature and reviewed by clinicians [35]. The full list of diagnosis codes used to identify delirium events is provided in Supplementary Table S3.

### 2.3. Conceptual Framework

Covariate selection was guided by the Andersen Behavioral Model and prior delirium prediction literature [36,37]. The Andersen framework was used to organize factors that may be related to both CAB and delirium risk, including demographic characteristics, access-related factors, baseline health status, comorbidity burden, frailty, and medication exposures.

Because several medication classes are associated with delirium risk, opioid load and sedative load were included as covariates. These measures captured exposure to opioids, benzodiazepines, and other sedative medications. The opioid load was calculated by taking the average Morphine Milligram Equivalent (MME) based on the CMS oral MME conversion factors per day over the time period [38]. Opioid use was categorized as low dose (less than 50 MME daily) and high dose (50 or greater MME daily) [39,40]. The medications used to calculate sedative [41,42,43] and opioid load, including morphine milligram equivalent conversion factors, are listed in Supplementary Tables S4 and S5. To avoid double-counting medication exposure, medications appearing in more than one burden measure were counted only once in CAB calculations.

Additional measures of baseline health status included the Elixhauser comorbidity index, claims-based frailty index, and anticholinergic exposure during the 12-month baseline period [44,45]. Comorbid conditions that may influence anticholinergic prescribing were also included, including conditions for which anticholinergic medications may be indicated or contraindicated [46]. Supplementary Tables S6 and S7 provide the full list of comorbidities and diagnosis codes.

### 2.4. Statistical Analysis

Baseline characteristics were summarized across CAB categories using descriptive statistics. Continuous variables were compared using analysis of variance, and categorical variables were compared using chi-square tests. Kaplan–Meier curves were used to examine unadjusted time to delirium across low/no, moderate, and high CAB groups. The log-rank test was used to compare the survival curves for statistical differences, and the proportional hazards assumption for the Cox model was assessed using the Schoenfeld test, checking the correlation between scaled residuals and time. The inverse probability of treatment weighting (IPTW) was employed in this study to generate unbiased treatment estimates by balancing the differences between CAB groups through weighting. In this study, we generated weights by using a machine learning approach based on the -generalized boosted models, which have been demonstrated to perform better than traditional approaches.

The generalized boosted model propensity score weights were obtained using the *Toolkit for Weighting and Analysis of Nonequivalent Groups* (TWANG) package with *mnps* function [47]. The balance was visually assessed using the boxplot for PS overlap and statistically by calculating the absolute standardized mean differences (ASMDs). An ASMD ≤ 0.25 was considered as the threshold for acceptable balance [48]. The Cox proportional-hazards regression model with IPTW based on a generalized boosted model score was used to evaluate the risk of delirium from each burden group. This study also applied the covariate adjustment approach using multiple propensity score techniques as a sensitivity analysis [49]. All statistical analyses were conducted using SAS, version 9.4 [50] and R software (Version 4.2) [51], a two-sided *p*-value < 0.05 was considered statistically significant.

## 3. Results

**Figure 1:**
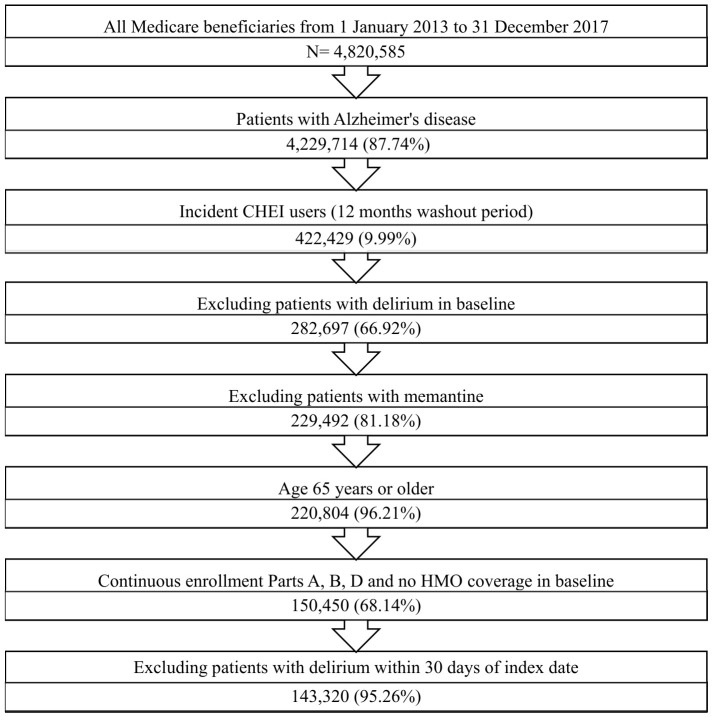
Attrition chart for developing older adult cohort with Alzheimer’s disease initiating on cholinesterase inhibitors.

The final cohort included 143,320 older adults with AD who met all eligibility criteria, as shown in Figure 1. At cohort entry, 62.74% of patients were classified as having low/no CAB, 16.14% as having moderate CAB, and 21.12% as having high CAB. The mean (SD) time to delirium event was 9 ± 5.16 months for the low/no burden group, 7 ± 6.77 months for the moderate burden, and 7 ± 6.02 months for the high burden group.

The cohort primarily consisted of women (66.40%), White (82.64%), and aged 85–94 years (41.69%) with a mean age of 82.07 ± 7.14 years. More than one-third (36.52%) had dual eligibility status, and 78.61% had a diagnosis of AD for 0–5 years. Dyslipidemia (69.76%), followed by depression (38.69%), muscle spasms/lower back pain (36.66%), and urinary tract infection (35.91%) were the most common comorbidities observed at baseline. The mean Elixhauser comorbidity score was found to be 6.13 ± 10.18, while the mean claims-based frailty index was 0.21 ± 0.06, with most patients in the prefrail category. Additionally, 23% of patients had a high sedative load, with most in the high-burden group. Over 18% of patients had a high opioid load, with most patients in the high-burden group (Table 1). See Table 1 and Supplementary Table S8 for all comorbidities evaluated in the baseline for the cohort.

**Figure 2:**
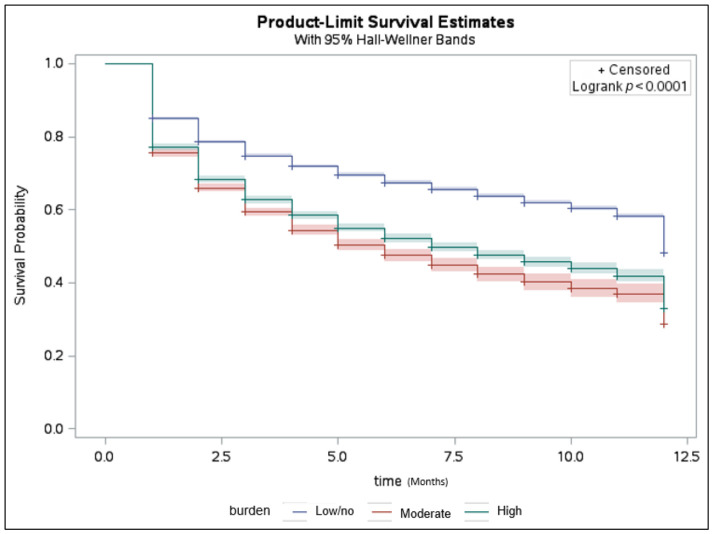
Kaplan–Meier plot for risk of delirium across three groups.

Overall, delirium diagnosis was observed in 19.11% of the cohort within one year of ChEI prescription. Figure 2 shows the Kaplan–Meier survival curves evaluating the risk of delirium among older adults with AD having different burdens. There was no differential censoring among the study groups (See Supplementary Table S9). The survival curve shows a significant crude association between the three burden categories and the risk of delirium (*p* = 0.0001) by log-rank test. Table 1 provides the distribution of baseline characteristics before and after PS adjustment using IPTW. After IPTW adjustment, measured baseline characteristics were well balanced in each of the categories. Both PS and generalized boosted model approaches helped to balance the baseline covariates (see Supplementary Material Figures S1–S4).

The primary Cox proportional hazards regression analysis with IPTW, with a low/no burden as the reference group, showed moderate burden (aHR 1.56; 95% CI, 1.52–1.61, *p* < 0.0001) and high burden (aHR 1.45; 95% CI, 1.42–1.49, *p* < 0.0001) were associated with an increased risk of delirium (See Table 2). The sensitivity analysis involving the Cox proportional hazards model with multiple PS adjustment methods was similar to those observed in the primary analyses. Moderate burden (aHR, 1.52; 95% CI, 1.48–1.56, *p* < 0.0001) and high burden (aHR, 1.30; 95% CI, 1.27–1.33, *p* < 0.0001) were associated with an increased risk of delirium as compared to low/no burden.

## 4. Discussion

In this Medicare cohort of older adults with AD who newly initiated ChEIs, high and moderate burdens were associated with about 50% increased risk of delirium compared to low/no burden. To the best of our knowledge, this study is among the first to examine delirium risk associated with patient-specific, dose-based CAB among older adults with AD initiating ChEIs. Our study also found that over one-third of older adults with AD had a moderate–high anticholinergic burden after initiating ChEIs. This finding highlights the substantial prevalence of anticholinergic exposure among older adults with AD despite recommendations to minimize the use of these medications in individuals with cognitive impairment. Given that anticholinergic burden is potentially modifiable, these findings underscore the importance of routine medication review and deprescribing efforts to reduce unnecessary anticholinergic exposure in patients receiving ChEI therapy. Since anticholinergic medications and ChEIs act in pharmacologically opposing directions, concurrent exposure may diminish the intended cholinergic benefit of ChEI therapy and increase vulnerability to adverse cognitive outcomes [18,19].

Our study found about a 50% higher risk of delirium with a moderate-to-high anticholinergic burden. The estimates of association for both moderate- and high-burden groups are in the same direction with an overlapping range of confidence intervals. Previous research has also found similar risk patterns for anticholinergic levels, possibly due to underlying effects and/or categorization [52]. Most importantly, studies have found similar results when studying the use of anticholinergics associated with an increased risk of delirium among older adults with dementia/AD using different methods and scales for measuring the burden but did not account for patient-specific dose and duration of medications prescribed and found the range of risk of delirium to be 1.5–4.09 [14,15]. A study utilizing the Delirium Drug Scale (DDS) found similar results for the risk of delirium [OR: 1.52 (weighted DDS score); OR: 1.20 (low exposure); OR: 1.60 (high exposure)] but differed in terms of the patient population [53]. The differences in effect size may be attributed to the study design, exposure measurement, covariates included in their study, and the clinical setting. These findings have important clinical implications. AD is itself a major risk factor for delirium, and delirium can accelerate cognitive and functional decline [5]. Anticholinergic exposure may further increase risk by compounding cholinergic deficits and counteracting the intended effects of ChEI therapy [18,19]. Since anticholinergic medications are potentially modifiable, routine medication review after ChEI initiation may help identify opportunities to reduce avoidable anticholinergic exposure. This is especially relevant for older adults with cognitive impairment, for whom anticholinergic medications are considered potentially inappropriate by guidance such as the American Geriatrics Society Beers Criteria, IPET, and STOPP/START criteria [54,55,56].

Strategies for preventing delirium can lower further adverse consequences, such as falls, fractures, functional deterioration, and mortality. Therefore, interventions that lessen or prevent delirium can mitigate or prevent long-term cognitive damage [5]. The Institute of Medicine-approved guidelines emphasized the value of multifaceted nonpharmacologic prevention strategies, healthcare professional education, medical evaluation of the etiology of delirium, maximizing nonopioid pain management, and avoiding high-risk medications [57]. In addition, multidisciplinary approaches involving pharmacist–physician interventions have demonstrated the potential to improve medication utilization for older patients who are at risk [58,59]. Widespread research has been conducted to offer evidence-based alternative medication treatments that help avoid the use of potent anticholinergic medications [60,61].

The strengths of the current study include the strong pharmacoepidemiologic design, methods, and analytical approaches. This is the first study to calculate CAB using patient-specific dosing, which is a more effective way to calculate the burden as we are accounting for the dose and duration of medication use. The results are highly generalizable due to national-level Medicare beneficiaries. Moreover, the propensity score approach based on the machine learning model helped mitigate the selection and confounding biases by controlling multiple covariates related to both the treatment groups and outcomes. Although anticholinergic medications have previously been associated with delirium [10,11,12,13,14,15,16] in older adults, evidence among patients with Alzheimer’s disease initiating cholinesterase inhibitors is limited. This study extends the existing literature by evaluating CAB in a large, nationally representative cohort of older adults with Alzheimer’s disease and quantifying its association with subsequent delirium risk.

Several limitations should be considered. Claims data do not include detailed clinical measures such as AD stage, cognitive test scores, laboratory values, genetic factors, or clinical rationale for prescribing. Therefore, AD severity could not be directly measured. We attempted to address this limitation by using proxy measures, including years since AD diagnosis, claims-based frailty, baseline anticholinergic burden, and exclusion of baseline memantine users. Delirium may be underdiagnosed or incompletely coded in claims data, which could lead to outcome misclassification. Pharmacy claims indicate that a medication was dispensed but do not confirm that the patient took the medication as prescribed. In addition, over-the-counter medications and medications not covered under Medicare Part D were not captured. The cohort was limited to fee-for-service Medicare beneficiaries with Part D coverage and no Medicare Advantage enrollment, which may limit generalizability to other populations or institutional settings. Finally, as with all observational studies, residual confounding may remain despite adjustment for measured covariates.

## 5. Conclusions and Implications

This study found that over one-third of patients with AD initiating ChEIs had moderate and high CAB. Most importantly, patients with AD having moderate and high CAB were associated with 56% and 45% increased risk of delirium compared to patients having low/no anticholinergic burden. This study helps recognize the importance of polypharmacy and medication management of anticholinergics to reduce the risk of delirium in AD. Therefore, concerted efforts are needed to reduce the CAB in older adults with AD by focusing on dose and duration, along with utilizing anticholinergic alternatives.

## Figures and Tables

**Table 1:** Baseline characteristics of older adults with Alzheimer’s disease initiating on cholinesterase inhibitors.

Characteristics	Overall N = 143,320	Low/No Burden (Score 1–10) n = 89,907 (62.73%)	Moderate Burden (Score 10–40) n = 23,138 (16.14%)	High Burden (Score > 40 or More) n = 30,275 (21.12%)	ASMD Before IPTW	ASMD After IPTW
Age groups (years)					0.148	0.008
Mean (±SD)	82.07 ± 7.09	81.75 ± 6.95	82.70 ± 7.26	81.55 ± 7.35		
65–74	21,275 (14.84%)	13,055 (14.52%)	3024 (13.07%)	5196 (17.16%)		
75–84	62,299 (43.47%)	40,122 (44.63%)	9319 (40.28%)	12,858 (42.47%)		
85 or above	59,746 (41.69%)	36,730 (40.85%)	10,795 (46.65%)	12,221 (40.37%)		
Sex					0.09	0.007
Female	95,163 (66.40%)	58,460 (65.02%)	15,872 (68.60%)	20,831 (68.81%)		
Male	48,157 (33.60%)	31,447 (34.98%)	7266 (31.40%)	9444 (31.19%)		
Race					0.081	0.008
White	118,444 (82.64%)	73,692 (81.96%)	19,337 (83.57%)	25,415 (83.95%)		
Black	13,019 (9.08%)	8201 (9.12%)	2078 (8.98%)	2740 (9.05%)		
Hispanic	4774 (3.33%)	3008 (3.35%)	775 (3.35%)	991 (3.27%)		
Asian	4266 (2.98%)	2635 (3.57%)	479 (2.33%)	552 (2.06%)		
Native American	500 (0.35%)	328 (0.36%)	80 (0.35%)	92 (0.30%)		
Other/Unknown	2317 (1.62%)	15,191 (1.77%)	312 (1.35%)	414 (1.37%)		
Dual eligibility					0.28	0.007
Yes	52,334 (36.52%)	28,592 (31.80%)	10,031 (43.35%)	13,711 (45.29%)		
No	90,986 (63.48%)	61,315 (68.20%)	13,107 (56.65%)	16,564 (54.71%)		
Years of diagnosis					0.118	0.006
0–5 years	112,671 (78.61%)	72,056 (80.15%)	17,867 (77.22%)	27,748 (75.14%)		
6–10 years	22,040 (15.38%)	12,953 (14.41%)	3724 (16.09%)	5363 (17.71%)		
11–15 years	7853 (5.48%)	4470 (4.97%)	1420 (6.14%)	1963 (6.48%)		
More than 15 years	756 (0.53%)	428 (0.48%)	127 (0.55%)	201 (0.66%)		
Baseline burden					0.39	0.007
Low/no	64,879 (45.27%)	58,152 (64.68%)	4791 (20.71%)	1936 (6.39%)		
Moderate	46,325 (32.32%)	22,857 (25.42%)	14,303 (61.82%)	9165 (30.27%)		
High	32,116 (22.41%)	8898 (9.90%)	4044 (17.48%)	19,174 (63.33%)		
High Sedative load	32,506 (22.68%)	16,306 (18.14%)	6380 (27.57%)	9820 (32.44%)	0.341	0.006
High Opioid load	26,772 (18.68%)	14,520 (16.15%)	4888 (21.13%)	7364 (24.32%)	0.21	0.006
Elixhauser comorbidity index score	6.13 ± 10.18	5.19 ± 9.43	7.51 ± 10.91	7.87 ± 11.32	0.18	0.005
Claims based frailty index	0.21 ± 0.06	0.20 ± 0.06	0.22 ± 0.06	0.23 ± 0.07	0.19	0.006
Robust	28,962 (20.21%)	21,677 (24.11%)	3376 (14.59%)	3909 (12.91%)		
Prefrail	71,097 (49.61%)	46,611 (51.84%)	10,829 (46.80%)	13,657 (45.11%)		
Mild frail	39,491 (27.55%)	20,261 (22.54%)	7998 (34.57%)	11,232 (37.10%)		
Moderate to severe frail	3770 (2.63%)	1358 (1.51%)	935 (4.04%)	1477 (4.88%)		
Comorbidities						
Surgery	1763 (1.23%)	954 (1.06%)	328 (1.42%)	481 (1.59%)	0.048	0.001
Renal insufficiency	26,425 (18.44%)	13,960 (15.53%)	5057 (21.86%)	7408 (24.47%)	0.231	0.007
Smoking	20,443 (14.26%)	11,426 (12.71%)	3627 (15.68%)	5390 (17.80%)	0.146	0.003
Vision & hearing impairment	7779 (5.43%)	4676 (5.20%)	1380 (5.96%)	1723 (5.69%)	0.034	0.007
Coma/stupor/brain damage	1705 (1.19%)	855 (0.95%)	344 (1.49%)	506 (1.67%)	0.066	0.003
Osteoporosis	26,970 (18.82%)	16,649 (18.52%)	4547 (19.65%)	5774 (19.07%)	0.029	0.006
Cancer/ malignancy	654 (0.46%)	373 (0.41%)	119 (0.51%)	162 (0.54%)	0.018	0.005
Transient ischemia	8286 (5.78%)	4932 (5.49%)	1423 (6.15%)	1931 (6.38%)	0.038	0.004
Urinary tract infection	45,280 (35.91%)	24,962 (27.76%)	8423 (36.40%)	11,895 (39.29%)	0.248	0.008
Influenza	1180 (0.82%)	671 (0.75%)	221 (0.96%)	288 (0.95%)	0.023	0.002
Thiamin deficiency	126 (0.09%)	75 (0.08%)	28 (0.12%)	23 (0.08%)	0.015	0.009
Vit. B12 deficiency	5872 (4.10%)	3582 (3.98%)	939 (4.06%)	1351 (4.46%)	0.024	0.004
Pain	14,502 (10.12%)	7343 (8.17%)	2874 (12.42%)	4285 (14.15%)	0.198	0.007
Urinary retention	40,608 (28.33%)	22,462 (24.98%)	7079 (30.59%)	11,067 (36.55%)	0.257	0.005
Use of bladder catheter	115 (0.08%)	52 (0.66%)	25 (0.11%)	38 (0.13%)	0.024	0.002
Mechanical ventilation	1483 (1.03%)	684 (0.76%)	311 (1.34%)	488 (1.61%)	0.084	0.003
Substance Abuse	4539 (3.17%)	2463 (2.74%)	894 (3.86%)	1182 (3.90%)	0.067	0.004
Hospitalization	29,030 (20.34%)	15,133 (16.93%)	5731 (24.83%)	8166 (27.01%)	0.252	0.003
Diagnosis (Negatively related-clinically exacerbated)						
Syncope	12,979 (9.06%)	7637 (8.49%)	2266 (9.79%)	3076 (10.16%)	0.058	0.002
Chronic seizures/epilepsy	6985 (4.87%)	3963 (4.41%)	1160 (5.01%)	1862 (6.15%)	0.081	0.004
Falls	15,145 (10.57%)	8368 (9.31%)	2830 (12.23%)	3947 (13.04%)	0.121	0.002
Fractures	13,302 (9.28%)	7511 (8.35%)	2573 (11.12%)	3218 (10.63%)	0.095	0.005
Pneumonia	10,438 (7.28%)	4928 (5.48%)	2254 (9.74%)	3256 (10.75%)	0.203	0.003
Hyperthyroidism	6505 (4.54%)	3804 (4.23%)	1128 (4.88%)	1573 (5.20%)	0.046	0.002
Heart failure	32,848 (22.92%)	15,136 (16.84%)	7009 (30.29%)	10,703 (35.35%)	0.441	0.007
Dyslipidemia	99,984 (69.76%)	61,606 (68.52%)	16,280 (70.36%)	22,098 (72.99%)	0.097	0.012
Narrow angle glaucoma	22,911 (15.99%)	14,665 (26.31%)	3573 (15.44%)	4673 (15.44%)	0.024	0.005
Myasthenia gravis	271 (0.19%)	152 (0.17%)	50 (0.22%)	69 (0.23%)	0.014	0.001
Myocardial infarction	3228 (2.25%)	1585 (1.76%)	690 (2.98%)	953 (3.15%)	0.093	0.004
Stroke	18,831 (13.14%)	10,763 (11.97%)	3368 (14.56%)	4700 (15.52%)	0.105	0.005
Dysrhythmia	46,197 (32.23%)	23,934 (26.62%)	9286 (40.13%)	12,977 (42.86%)	0.348	0.009
Chronic constipation	20,815 (14.52%)	11,562 (12.86%)	3796 (16.41%)	5457 (18.02%)	0.147	0.003
Benign prostatic hyperplasia	5387 (3.76%)	3223 (3.58%)	896 (3.87%)	1268 (4.19%)	0.032	0.009
Parkinson’ s disease	9324 (6.51%)	5364 (5.97%)	1498 (6.47%)	2462 (8.13%)	0.088	0.011
Lewy body disease	6563 (4.58%)	3900 (4.34%)	1098 (4.75%)	1565 (5.17%)	0.04	0.003
Diagnosis (Positively related-necessitates ACH)						
Behavioral and psychological symptoms (BPSD)	20,167 (14.07%)	10,037 (11.16%)	4338 (18.75%)	5792 (19.13%)	0.229	0.007
Mood disorders	17,711 (12.36%)	8542 (9.50%)	3423 (14.79%)	5746 (18.98%)	0.288	0.006
Anxiety	25,638 (17.89%)	12,967 (14.42%)	5043 (21.80%)	7628 (25.20%)	0.281	0.007
Urinary incontinence	16,608 (11.59%)	8806 (9.79%)	2579 (11.15%)	5223 (17.25%)	0.233	0.007
Muscle spasm/lower back pain	52,546 (36.66%)	31,133 (34.63%)	8853 (38.26%)	12,560 (41.49%)	0.024	0.011
Depression	55,451 (38.69%)	29,433 (32.74%)	10,462 (45.22%)	15,556 (51.38%)	0.383	0.01
Abnormal involuntary movements	34,895 (24.35%)	19,471 (21.66%)	6366 (27.51%)	9058 (29.92%)	0.193	0.006
Gastrointestinal reflux disease	36,761 (25.65%)	19,879 (22.11%)	6868 (29.68%)	10,014 (33.08%)	0.251	0.009
Insomnia	12,780 (8.92%)	6773 (7.53%)	2480 (10.72%)	3527 (11.65%)	0.144	0.005
Irritable bowel disease	3461 (2.41%)	1877 (2.09%)	623 (2.69%)	961 (3.17%)	0.071	0.004
Neuropathic pain	2394 (1.67%)	1405 (1.56%)	388 (1.68%)	601 (1.99%)	0.033	0.002
Secondary Parkinsonism	1069 (0.75%)	592 (0.66%)	141 (0.61%)	336 (1.11%)	0.058	0.005
Claims based frailty index	0.21 ± 0.06	0.20 ± 0.06	0.22 ± 0.06	0.23 ± 0.07	0.19	0.006
Robust	28,962 (20.21%)	21,677 (24.11%)	3376 (14.59%)	3909 (12.91%)		
Prefrail	71,097 (49.61%)	46,611 (51.84%)	10,829 (46.80%)	13,657 (45.11%)		
Mild frail	39,491 (27.55%)	20,261 (22.54%)	7998 (34.57%)	11,232 (37.10%)		
Moderate to severe frail	3770 (2.63%)	1358 (1.51%)	935 (4.04%)	1477 (4.88%)		

ASMD: absolute standardized mean differences. IPTW: inverse probability of treatment weighting.

**Table 2:** Cox-proportional hazards model for the risk of delirium among older adults with Alzheimer’s disease initiating on cholinesterase inhibitors.

Anticholinergic Burden	Unadjusted Analysis		Adjusted Analysis *	
	Unadjusted HR (95%CI)	*p*-Value	Adjusted HR (95%CI)	*p*-Value
Low/no burden	Reference		Reference	
Moderate burden	1.76 (1.71–1.80)	<0.0001	1.56 (1.52–1.61)	<0.0001
High burden	1.55 (1.52–1.59)	<0.0001	1.45 (1.42–1.49)	<0.0001

* Adjusted analysis stands for IPT weighted analysis.

## Data Availability

The data presented in this study are available on request from the corresponding author due to data use agreements and other licenses of Centers for Medicare & Medicaid Services.

## Supplementary Materials

The following supporting information can be downloaded at https://www.mdpi.com/article/10.3390/pharmacy14040089/s1. Table S1: List of drugs with ACB score and defined daily dose for calculating patient-specific dosing; Table S2: Calculation for cumulative anticholinergic burden; Table S3: Codes for identification of delirium; Table S4: Drugs included under the sedative load model; Table S5: Drugs included to calculate opioid load; Table S6: Comorbidities affecting anticholinergic burden; Table S7: ICD codes used for identifying comorbidities; Table S8: Baseline characteristics of older adults with Alzheimer’s disease initiating on cholinesterase inhibitors; Table S9: Extent and time to censoring across study groups; Figure S1: Overlap of propensity scores in each exposure group; Figure S2: Balancing using generalized boosting method for multiple propensity scores; Figure S3: Overlap of propensity scores calculated for multiple propensity score weighting using generalized boosting methods; Figure S4: Log negative Log plot; Strobe Checklist for cohort studies.
