# Supplementary material for: Cumulative Anticholinergic Burden and Risk of Delirium Among Older Adults with Alzheimer’s Disease
Source: Pharmacy (Basel). 2026 Jun 23;14(4):89. doi: 10.3390/pharmacy14040089 (PMC13414801; doi:10.3390/pharmacy14040089)
Supplement: Supplementary file 1 [file pharmacy-14-00089-s001.zip › pharmacy-4348713-supplementary.pdf]

**RISK OF DELIRIUM ASSOCIATED WITH CUMULATIVE ANTICHOLINERGIC BURDEN IN OLDER ADULTS WITH ALZHEIMER'S DISEASE**

**SUPPLEMENTARY MATERIAL**

**Supplementary Table S1. List of drugs with ACB score and defined daily dose for calculating patient-specific dosing.**

| Drugs | ACB Score | Defined daily dose (DDD) | Unit |
|---|---|---|---|
| Trimeprazine | 1 | 30 | mg |
| Alverine | 1 | 60 | mg |
| Asenapine | 1 | 20 | mg |
| Aripiprazole | 1 | 15 | mg |
| Alprazolam | 1 | 1 | mg |
| Atenolol | 1 | 75 | mg |
| Bupropion | 1 | 0.3 | gm |
| Captopril | 1 | 50 | mg |
| Cetirizine | 1 | 10 | mg |
| Chlorthalidone | 1 | 25 | mg |
| Clidinium | 1 | 0.64 | mg |
| Cimetidine | 1 | 0.8 | mg |
| Clorazepate | 1 | 20 | mg |
| Codeine | 1 | 0.1 | gm |
| Colchicine | 1 | 1 | mg |
| Desloratadine | 1 | 5 | mg |
| Diazepam | 1 | 10 | mg |
| Digoxin | 1 | 0.25 | mg |
| Dipyridamole | 1 | 0.4 | gm |
| Disopyramide | 1 | 0.4 | gm |
| Fentanyl | 1 | 0.6 | mg |
| Furosemide | 1 | 40 | mg |
| Fluvoxamine | 1 | 0.1 | gm |
| Haloperidol | 1 | 8 | mg |
| Hydralazine | 1 | 0.1 | gm |
| Hydrocortisone | 1 | 30 | mg |
| Iloperidone | 1 | 18 | mg |
| Isosorbide | 1 | 40 | mg |
| Levocetirizine | 1 | 5 | mg |
| Loperamide | 1 | 10 | mg |
| Loratadine | 1 | 10 | mg |

| | | | |
|---|---|---|---|
| Metoprolol | 1 | 0.15 | gm |
| Morphine | 1 | 0.1 | gm |
| Nifedipine | 1 | 30 | mg |
| Paliperidone | 1 | 6 | mg |
| Prednisone | 1 | 10 | mg |
| Quinidine | 1 | 1.2 | gm |
| Ranitidine | 1 | 0.3 | gm |
| Risperidone | 1 | 5 | mg |
| Theophylline | 1 | 0.4 | gm |
| Trazodone | 1 | 0.3 | gm |
| Triamterene | 1 | 0.1 | gm |
| Venlafaxine | 1 | 0.1 | gm |
| Warfarin | 1 | 7.5 | mg |
| Amantadine | 2 | 0.2 | gm |
| Belladonna alkaloids | 2 | 1 | mg |
| Carbamazepine | 2 | 1 | gm |
| Cyclobenzaprine | 2 | 5 | mg |
| Cyproheptadine | 2 | 12 | mg |
| Acetaminophen-Codeine | 2 | 30 | mg |
| Loxapine | 2 | 9.1 | mg |
| Meperidine | 2 | 50 | mg |
| Levomepromazine | 2 | 0.3 | gm |
| Molindone | 2 | 50 | mg |
| Nefopam | 2 | 30 | mg |
| Oxcarbazepine | 2 | 1 | gm |
| Pimozide | 2 | 4 | mg |
| Pethidine | 2 | 0.4 | gm |
| Amitriptyline | 3 | 75 | mg |
| Amoxapine | 3 | 0.15 | gm |
| Atropine | 3 | 1.5 | mg |
| Benztropine | 3 | 2 | mg |
| Brompheniramine | 3 | 24 | mg |
| Carbinoxamine | 3 | 16 | mg |
| Chlorpheniramine | 3 | 6 | mg |
| Chlorpromazine | 3 | 0.3 | gm |
| Clemastine | 3 | 2 | mg |
| Clomipramine | 3 | 0.1 | gm |
| Clozapine | 3 | 0.3 | gm |
| Darifenacin | 3 | 7.5 | mg |
| Desipramine | 3 | 0.1 | gm |
| Dicyclomine | 3 | 80 | mg |

| | | | |
|---|---|---|---|
| Dimenhydrinate | 3 | 50 | mg |
| Diphenhydramine | 3 | 0.2 | gm |
| Doxepin | 3 | 0.1 | gm |
| Fesoterodine | 3 | 4 | mg |
| Flavoxate | 3 | 0.8 | gm |
| Hydroxyzine | 3 | 75 | mg |
| Hyoscyamine | 3 | 1.2 | mg |
| Imipramine | 3 | 0.1 | gm |
| Meclizine | 3 | 25 | mg |
| Methocarbamol | 3 | 3 | gm |
| Nortriptyline | 3 | 75 | mg |
| Olanzapine | 3 | 10 | mg |
| Orphenadrine | 3 | 0.12 | gm |
| Oxybutynin | 3 | 15 | mg |
| Paroxetine | 3 | 20 | mg |
| Perphenazine | 3 | 30 | mg |
| Procyclidine | 3 | 25 | mg |
| Promazine | 3 | 0.3 | gm |
| Promethazine | 3 | 25 | mg |
| Propantheline | 3 | 60 | mg |
| Propiverine | 3 | 30 | mg |
| Pyrilamine | 3 | 0.2 | gm |
| Quetiapine | 3 | 0.4 | gm |
| Scopolamine | 3 | 0.9 | mg |
| Solifenacin | 3 | 5 | mg |
| Thioridazine | 3 | 0.3 | gm |
| Tolterodine | 3 | 4 | mg |
| Trifluoperazine | 3 | 20 | mg |
| Trihexyphenidyl | 3 | 10 | mg |
| Trimipramine | 3 | 0.15 | gm |
| Trospium | 3 | 40 | mg |

Source: ACB SCALE[1]; DDD[2, 3]

**Supplementary Table S2. Calculation for cumulative anticholinergic burden.**

| |
|---|
| **1) Exposure measure**: Total medication dose for each prescription fill by multiplying the tablet strength by number of tablets dispensed. |
| **2) Standardized daily dose (SDD):** Divide by the minimum effective dose per day recommended for use in older adults.<br>$$\text{Standardized daily dose (SDD)} = \frac{\text{No. of daily units * unit dose}}{\text{Defined daily dose (DDD)}}$$ |
| 3) **Drug-specific standardized Daily ACH Exposure (SDACE):** Each SDD for each patient *ACB scale score.<br>For each participant, sum the SDD for all anticholinergic pharmacy fills during the exposure period to create a cumulative total standardized daily dose (SumSDACE). |
| 4) **Cumulative exposure** for each patient obtained by summing daily SumSDACE values. |

**Supplementary Table S3. Codes for identification of delirium.**

| | |
|---|---|
| **ICD 9 codes[4]** | 293, 293.1, 292.81, 290.11, 290.3, 290.41, 291.0, 293.9, 780.09, 290.12, 290.13, 290.43, 292.11, 292.12, 292.2, 780.02, 290.2, 290.42, 348.3, 348.31, 348.39, 349.82, 780.97 |
| **ICD 10 codes[5]** | F05, F10.121, F10.221, F10.231, F10.921, F11.121, F11.221, F11.921, F12.121, F12.221, F12.921, F13.121, F13.221, F13.231, F13.921, F13.931, F14.121, F14.221, F14.921, F15.121, F15.221, F15.921, F16.121, F16.221, F16.921, F18.121, F18.221, F18.921, F19.121, F19.221, F19.231, F19.921, F19.931, G92*, G93.40, G93.41, G93.49 |

**Supplementary Table S4. Drugs included under the sedative load model [6-8].**

| Category | Drugs* |
|---|---|
| Score 2 : Primary Sedatives | |
| Antidepressants | Dothiepin, Mianserine, Moclobemide, Quinupramine, |
| Barbiturates | Pentobarbital, Phenobarbital, Thiamylal, Thiopental |
| Benzodiazepines | Bromazepam, Brotizolam, Clobazam, Clonazepam, Clotiazepam, Estazolam, Ethyl Loflazepate, Etizolam, Flunitrazepam, Flurazepam, Lorazepam, Mexazolam, Midazolam, Pinazepam, Temazepam, Tofisopam, Triazolam |
| General Anesthetics | Etomidate, Ketamine, Propofol |
| Other Anxiolytics | Buspirone, Tandospirone |
| Other Hypnotic & Sedatives | Chloral Hydrate, Dichloralphenazone |
| Traditional Antipsychotics | Bromperidol, Chlorprothixene, Droperidol, Lithium, Mesoridazine, Nemonapride, Sulpiride, Thiothixene, Tiapride, Zotepine, Zuclopenthixol |
| Z-Drugs | Zolpidem, Zopiclone |
| | |
| Score 1: Drugs with Sedation as a Prominent Side Effect or Preparations with a Sedating Component | |
| Antidepressants | Citalopram, Duloxetine, Hyperici herba, Escitalopram, Fluoxetine, Medifoxamine fumarate, Milnacipran, Mirtazapine, Nefazodone, Sertraline, Tianeptine, Toloxatone, Venlafaxine |
| Antiepileptics | Fosphenytoin, Gabapentin, Lamotrigine, Levetiracetam, Phenytoin, Pregabalin, Primidone, Topiramate, Valproic Acid, Vigabatrin, Zonisamide |
| Antimigraine | Almotriptan, Ergotamine, Frovatriptan, Naratriptan, Sumatriptan, Zolmitriptan |
| Antiparkinson Drugs | Benzatropine, Biperiden, |
| Antivertigo & | Betahistine, Cinnarizine, Difenidol, Dimenhydrinate |

| | |
|---|---|
| Antiemetics | |
| Atypical Antipsychotics | Amisulpride, Blonanserin, Ziprasidone |
| Central Muscle Relaxants | Afloqualone, Baclofen, Carisoprodol, Chlormezanone, Chlorphenesin, Chlorzoxazone, Cyclobenzaprine, Eperisone, Methocarbamol, Orphenadrine, Pridinol, Thiocolchicoside, Tizanidine, Tolperisone |
| Opioids | Alfentanil, Buprenorphine, Butorphanol, Dihydrocodeine, Hydrocodone, Hydromorphone, Nalbuphine, Naloxone, Pentazocine, Remifentanil, Sufentanil, Tramadol |
| Old Antihistamines | Buclizine, Chlorcinnazine, Dexbrompheniramine, Diphenylpyraline, Doxylamine, Homochlorcyclizine, Mequitazine, Oxatomide, Oxomemazine, Pheniramine, Piprinhydrinate, Triprolidine |
| Prokinetics | Metoclopramide |
| Respiratory | Benproperine, Benzonatate, Bromhexine, Dextromethorphan, Levodropropizine |

***after excluding the drugs considered in calculating ACH burden.**

**Supplementary Table S5. Drugs included to calculate opioid load [9].**

| **Drugs*** | **Morphine milligram equivalence conversion factor** |
|---|---|
| Butorphanol | 7 |
| Dihydrocodeine | 0.25 |
| Hydrocodone | 1 |
| Hydromorphone | 4 |
| Oxycodone | 1.5 |
| Pentazocine | 0.37 |
| Tramadol | 0.1 |
| Tapentadol | 0.4 |
| Methadone | 8 |
| Opium | 1 |

*After excluding the drugs considered in calculating ACH burden.

**The opioid load was calculated by taking the average Morphine Milligram Equivalent (MME) based on the CMS oral MME conversion factors per day over the time period. [10] Opioid use was categorized as low dose (less than 50 MME daily) and high dose (50 or greater MME daily). [9, 11]

**Supplementary Table S6. Comorbidities affecting anticholinergic burden.**

| Diagnoses clinically needing ACH prescription | Diagnoses clinically exacerbated with ACH prescription |
|---|---|
| Abnormal involuntary movements | Acute myocardial infarction |
| Anxiety | Benign prostatic hyperplasia |
| Behavioral psychological symptoms of dementia (BPSD) | Cardiac arrhythmias |
| Bipolar disorder | Chronic constipation |
| Depression | Chronic seizures/epilepsy |
| Gastroesophageal reflux disease (GERD) | Dyslipidemia |
| Insomnia | Dysrhythmia |
| Irritable bowel syndrome (IBS) | Falls |
| Mood disorders | Fractures |
| Muscle spasm/ lower back pain | Heart Failure |
| Neuropathic pain | Hyperthyroidism |
| Secondary Parkinson’s disease | Myasthenia gravis |
| Urinary incontinence | Myocardial infarction |
| | Narrow-angle glaucoma |
| | Lewy body disease |
| | Parkinson’s disease |
| | Pneumonia |
| | Stroke |
| | Syncope |
| | |

**Supplementary Table S7. ICD codes used for identifying comorbidities.**

| Comorbidities | ICD-9 and ICD-10 codes |
|---|---|
| **Gastroesophageal reflux disease** | 530.81, K21.9 |
| **Thyroid Disorder** | 242.9, 242.90, 242.91, 242.0, 242.01, 245.0, 245.1, 245.2, 245.3, 245.4, 245.8, 245.9, 246.0, 246.1, 246.2, 246.3, 246.8, 246.9, E07.9, E05.01, E05.00, E05.90, E05.91 |
| **Urinary Incontinence** | 307.6, 625.6, 788.30, 788.31, 788.33, 788.37, 788.38, 788.39,788.91, 788.3, |

| | |
|---|---|
| | R32, F98.0, N39.3, N39.41, N39.46, N39.45, N39.490, N39.491, N39.492, N39.498, N39.81 |
| **Syncope** | 780.2, R55 |
| **Stroke** | 346.60, 346.61,346.62, 346.63, 430, 431, 432.0, 432.1, 432.9, 433.01, 433.11, 433.21 , 433.31 ,433.81, 433.91, 434.0, 434.00, 434.01, 434.1, 434.10, 434.11, 434.9, 434.90, 434.91, 436, 438, 438.0, 438.10, 438.11, 438.12, 438.13, 438.14, 438.19 , 438.20, 438.21, 438.22, 438.40, 438.41, 438.42, 438.50, 438.51, 438.52, 438.53, 438.6, 438.7, 438.81, 43882,43883,43884 ,43885 , 43889 , 4389, G43.609, G43.619, G43.601, G43.611, I60.9, I61.9, I62.1, I62.00, I62.9, I65.1, I63.22, I63.00, I65.29, I63.139, I63.239, I65.09, I63.019, I63.119, I63.219, I63.9,I65.8, I63.59, I65.9, I63.20 |
| **Seizure** | 345, 345.0, 345.00, 345.01, 345.1, 345.10, 345.11, 345.2, 345.3, 345.4, 345.40, 345.41, 345.5, 345.50, 345.51, 345.6, 345.60, 345.61, 345.7, 345.71, 345.8, 345.80, 345.81, 345.9, 345.90, 345.91, 780.3, 780.31, 780.32, 780.33, 780.39<br>R56.00, R56.01, R56.1, R56.9, G40.A01, G40.A09, G40.A11, G40.A19, G40.401, G40.309, G40.311, G40.409, G40.411, G40.419, G40.301, G40.201, G40.209, G40.211, G40.219, G40.101, G40.109, G40.111, G40.119, G40.821, G40.822, G40.802, G40.804, G40.823, G40.824, G40.501, G40.509, G40.901, G40.909, G40.911, G40.919 |
| **Myocardial Infarction** | 410.0, 410.00, 410.01, 410.02, 410.1, 410.10, 410.11, 410.12, 410.2, 410.20, 410.21, 410.22, 410.3, 410.30, 410.31, 410.32, 410.4, 410.40, 410.41 ,410.42, 410.5, 410.50, 410.51, 410.52, 410.6, 410.60 ,410.61 , 410.62, 410.7, 410.71, 410.72, 410.8, 410.80, 410.81, 410.82, 410.9, 410.90, 410.91, 410.92,<br>I21.09, I21.19, I21.11, I21.29, I21.4, I21.3,I21.9, I21.A1, I21.A9 |
| **Impaired Movements** | R26, R29.8,781.2, V57.81,781.99, V46.3, Z99.3, 781.0, R25.0, R25.1, R25.2, R25.3, R25.9, R29.6, 781.3<br>HCPCS codes - E1050-E1093, E1100-E1110, E1130-E1161, E1170-E1200, E1220-E1239; E1240-E1270; E1280-E1298; E1280-E1298 |
| **Neuropathic pain** | 349.9,729.2, M79.2 |
| **Inflammatory bowel syndrome** | 564.1, K58, K58.9 |
| **Insomnia** | 780.52, G47.00 |
| **Dystonia** | 333.72, 333.89, G24.02, G24.9 |
| **Secondary Parkinson** | 332.1, G21.11, G21.19, G21.8 |
| **Heart Failure** | 428, 428.1, 428.2, 428.3, 428.4. 428.9, 398, 402, 425,<br>I09.9, I11.0, I11.9 I13.0, I13.2, I25.5, I42.0, I42.5, I42.6, I42.7, I42.8, I429, I43, I50 |
| **Cardiac Arrhythmia** | 427, 427.1, 427.41, 427.42, 427.60, 427.81, 427.89, 427.9, 426.12<br>I44.1, I44.2, I44.3, I45.6, I45.9, I47, I48, I49, R00.0, R00.1, R00.8, T82.1, Z45.0, Z95.0 |
| **Depression** | 296, 309, 311, F20.4, F31.3, F31.4, F32, F33, F34.1, F41.2 |

| | |
|---|---|
| **Glaucoma** | 365.00, 365.01, 365.02, 365.03, 365.04, 365.05, 365.06, 365.10, 365.11, 365.12, 365.13 ,365.14, 365.15, 365.20, 365.21, 365.22, 365.23, 365.24, 365.31, 365.32, 365.41, 365.42, 365.43, 365.44, 365.51, 365.52, 365.59, 365.60, 365.61, 365.62, 365.63, 365.64, 365.65, 365.70, 365.71, 365.72, 365.73, 365.74, 365.81, 368.52 ,356.838, 365.89, 365.9<br>H40.009, H40.019, H40.039, H40.049, H40.059, H40.029, H40.069, H40.10X0, H40.10X1, H4010X2, H40.10X3, H40.10X4, H40.1110, H40.1111, H40.1112, H40.1113, H40.1114, H40.1120, H40.1121, H40.249, H40.1490, H40.219, H40.2290, H40.1122, H40.1123, H40.1124, H40.1130, H40.1131, H40.1132, H40.1133, H40.1134, H40.1190, H40.1191, H40.1192, H40.1193, H40.1194, H40.1290, H40.1390, Q150, H40.159, H40.20X0, H4020X1, H40.20X2, H40.20X3, H40.20X4, H40.239, H40.9, H42, H40.50X0, H40.30X0, H40.40X0, H40.829, H40.839, H40.819, H40.89, H40.60X0 |
| **Parkinson's Disease** | 332.0, G20 |
| **Lewy body Dementia** | 331.82, G31.83 |
| **Benign prostatic hyperplasia** | 600.90, 600.0, 600.91, N40.0, N40, N40.1 |
| **Backpain** | 720.1, 720.2, 720.81, 720.89, 720.9, 721.0, 721.1, 721.2, 721.3, 721.41, 721.42, 721.5, 721.6, 721.7, 721.8, 72190, 721.91, 722.0, 722.10, 722.11, 722.2, 722.30, 722.31, 722.32, 722.39, 722.4, 722.51, 722.52, 722.6, 722.70, 722.71, 722.72, 722.73, 722.80, 722.81, 722.82, 722.83, 722.90, 722.91, 722.92, 722.93, 723.0, 723.1, 723.2, 723.3, 723.4, 723.5, 723.6, 723.7, 723.8, 723.9, 724.00, 724.01, 724.02, 724.09, 724.1, 724.2, 724.3, 724.4, 724.5, 724.6,724.70, 724.71, 724.79, 7248, 724.9<br>M45.9, M43.8X9, M53.9, M54.08, M48.04, M48.061, M48.08, M48.062, M54.6, M54.14, M54.15, M51.26, M51.27, M542, M54.16, M54.17, M54.89, M54.9, M53.3, M46.40, M51.9, M53.1, M67.88, M53.82, M53.2X8 |
| **Fracture** | 820.00, 820.01, 820.02, 820.03, 820.09, 820.10, 820.11, 820.12, 820.13, 820.19, 820.20, 820.21, 820.22, 820.30, 820.31, 820.32, 820.8, 820.9, 905.3, V54.13, V54.23, 820, 821, 821.0, 821.00, 821.01, 821.1, 821.10, 821.11, 821.2, 810, 821.20, 821.21, 821.22, 82123, 821.29, 821.3, 821.30, 821.31, 821.32, 821.33, 821.39, 733.10, 733.8, 733.93, 733.94, 733.95, 733.96, 733.97,733.98, 805.4, 806.4, 839.20<br>S32, S33, S42, S43, S52, S53, S62, S63, S72, S73, M80, M81, M84.350A, S72.019A, S72.109B, S72.109C, S72.019B, S72.109A, S72.019C, S72.009B, S72.009C, S72.099B, S72.099C, S72.099A, S72.23XC, S33.101A, S32.009A, S34.109A, S34.119A, S34.129A, S32.019A, S34.101A, S34.111A, S34.121A, S32.029A, S34.102A, S34.112A, S34.122A, S32.039A, S34.103A, S34.113A, S34.123A, S32.049A, S34.104A, S34.114A, S34.124A, S32.059A, S34.105A, S34.115A, S34.125A |

| | |
|---|---|
| **Falls** | E880.0, E880.1, E880.9, E881.0, E881.1, E882, E883.0, E883.1, E883.2, E883.9, E884.0, E884.1, E884.2, E884.3, E884.4, E884.5, E884.6, E88.49, E885, E885.0, E885.1, E885.2, E885.3, E885.4, E885.9, E886.0, E886.9, E888, E888.0, E888.1, E888.8, E888.9, E968.1, E987.0, E987.1, E987.2, E987.9<br>W01, W00, W03.XXXA, W17.0XXA, W17.1XXA, V00.321A, V00.312A, W18.49XA, Y30.XXXA, W10.8XXA, W10.1XXA, W09.8XXA, W07.XXXA, W14.XXXA, W17.81XA, W17.89XA, W06.XXXA, W18.11XA, V00.388A |
| **Pneumonia** | 480.0, 480.1, 480.2, 480.3, 480.8, 480.9, 481, 482.0, 482.2, 482.1, 482.30, 482.31, 482.32, 482.39, 482.40, 482.41, 482.42, 482.49, 482.81, 482.82, 482.83, 482.84, 482.89, 482.9, 483.0, 483.1, 483.8, 484.1, 484.3, 484.5, 484.6, 484.7, 484.8, 485, 486, 5070, J18, B96.0, J12, J15, J16, J12, J13, J14, J17 |
| **Hyperlipidemia** | 272.0, 272.1, 272.2, 272.3, 272.4, E78.00, E78.01, E78.2, E78.3, E78.4, E78.5 |
| **Anxiety** | 293.84, 300.00, 300.01, 300.02, 300.09, 300.10, 300.20, 300.21, 300.22, 300.23, 300.29, 300.3, 300.5, 300.89, 300.9, 308.0, 308.1, 308.2, 308.3, 308.4, 308.9, 309.81<br>F06.4, F41.1, F41.9 |
| **Mood Disorder** | 293.83, 296.00, 296.01, 296.02, 296.03, 296.04, 296.05, 296.06, 296.10, 296.11, 296.12, 296.13, 296.14, 296.15, 296.16, 296.20, 296.21, 296.22, 296.23, 296.24, 296.25, 296.26, 296.30, 296.31, 296.32, 296.33, 296.34, 296.35, 296.36, 296.40, 296.41, 296.42, 296.43, 296.44, 296.45, 296.46, 296.50, 296.51, 296.52, 296.53, 296.54, 296.55, 296.56 , 296.60, 296.61, 296.62, 296.63, 296.64, 296.65, 296.66, 296.7, 296.80, 296.81, 296.82, 296.89, 296.90, 296.99, 300.4, 311<br>F06.30, F30.10, F30.11, F30.4, F31.62, F06.30, F31.63, F31.78, F30.3, F31.60, F31.32, F30.8, F39, F31.81, F31.2, F32.4, F32.1, F33.2, F33.42 |
| **Chronic Constipation** | 564.00, 564, K59.09, K59 |
| **Myasthenia Gravis** | 358.0, G70.0 |
| **Behavioral and psychological symptoms of dementia (BPSD)** | |
| **Agitation** | 307.9, R45.1 |
| **Aggression** | 312.9, 312.01, 312.10, 312.89, R45.6, F91.8 |
| **Psychosis** | 293.82, 293.81, 297.1, 297.2, 297.0, 297.3, 297.8, 297.9, 298.0, 298.1, 298.2 , 298.3, 298.4, 298.8, 298.9, 780.1, 368.16, 293.81, 293.82, 290.8, 290.9, 295.00, 295.01, 295.02, 295.03, 295.04, 295.05, 295.10, 295.11, 295.12, 295.13, 295.14, 295.15, 295.20, 295.21, 295.22, 295.23, 295.24, 295.25, 295.30, 295.31, 295.32, 295.33, 295.34, 295.35, 295.40, 295.41, 295.42, 295.43, 295.44, 295.45, 295.50 ,295.51, 295.52, 295.53, 295.54, 295.55, 295.60, 295.61, 295.62, 295.63, 295.64, 295.65, 295.70, 295.71, 295.72, 295.73, 295.74, 295.75, 295.80, 295.81, 295.82, 295.83, 295.84, 295.85, 295.90, 295.91, 295.92, 295.93, 295.94, 295.95 |

| | |
|---|---|
| | F06.0, F06.2, F20, F23, F24, F28, F29, F30.2, F31.5, F31.64, F32.3, F33.3, F25.1, F20.0, F20.5, F20, F20.0, F20.2, F20.81, F20.89, F25.9, F20.9 |
| **Wandering** | Z91.83, V40.31 |
| **Behavioral Symptoms** | 294.11, 294.21, F01.51, F02.81, F03.91 |
| | |

**Supplementary Table S8. Baseline characteristics of older adults with Alzheimer's disease initiating on cholinesterase inhibitors.**

| **Characteristics** | **Overall N=143,320** | **Low-No burden (Score 1-10) n= 89,907 (62.73%)** | **Moderate burden (Score 10-40) n= 23,138 (16.14%)** | **High burden (>40 or more) n= 30,275 (21.12%)** | **ASMD before IPTW** | **ASMD after IPTW** |
|---|---|---|---|---|---|---|
| **Comorbidities** | | | | | | |
| Surgery | 1763 (1.23%) | 954 (1.06%) | 328 (1.42%) | 481 (1.59%) | 0.048 | 0.001 |
| Renal insufficiency | 26425 (18.44%) | 13960 (15.53%) | 5057 (21.86%) | 7408 (24.47%) | 0.231 | 0.007 |
| Smoking | 20443 (14.26%) | 11426 (12.71%) | 3627 (15.68%) | 5390 (17.80%) | 0.146 | 0.003 |
| Vision & hearing impairment | 7779 (5.43%) | 4676 (5.20%) | 1380 (5.96%) | 1723 (5.69%) | 0.034 | 0.007 |
| Coma/stupor/ brain damage | 1705 (1.19%) | 855 (0.95%) | 344 (1.49%)) | 506 (1.67%) | 0.066 | 0.003 |
| Osteoporosis | 26970 (18.82%) | 16649 (18.52%) | 4547 (19.65%) | 5774 (19.07%) | 0.029 | 0.006 |
| Cancer/ malignancy | 654 (0.46%) | 373 (0.41%) | 119 (0.51%) | 162 (0.54%) | 0.018 | 0.005 |
| Transient ischemia | 8286 (5.78%) | 4932 (5.49%) | 1423 (6.15%) | 1931 (6.38%) | 0.038 | 0.004 |
| Urinary tract infection | 45280 (35.91%) | 24962 (27.76%) | 8423 (36.40%) | 11895 (39.29%) | 0.248 | 0.008 |
| Influenza | 1180 (0.82%) | 671 (0.75%) | 221 (0.96%) | 288 (0.95%) | 0.023 | 0.002 |
| Thiamin deficiency | 126 (0.09%) | 75 (0.08%) | 28 (0.12%) | 23 (0.08%) | 0.015 | 0.009 |
| Vit. B12 deficiency | 5872 (4.10%) | 3582 (3.98%) | 939 (4.06%) | 1351 (4.46%) | 0.024 | 0.004 |

| | | | | | | |
|---|---|---|---|---|---|---|
| Pain | 14502 (10.12%) | 7343 (8.17%) | 2874 (12.42%) | 4285 (14.15%) | 0.198 | 0.007 |
| Urinary retention | 40608 (28.33%) | 22462 (24.98%) | 7079 (30.59%) | 11067 (36.55%) | 0.257 | 0.005 |
| Use of bladder catheter | 115 (0.08%) | 52 (0.66%) | 25 (0.11%) | 38 (0.13%) | 0.024 | 0.002 |
| Mechanical ventilation | 1483 (1.03%) | 684 (0.76%) | 311 (1.34%) | 488 (1.61%) | 0.084 | 0.003 |
| Substance Abuse | 4539 (3.17%) | 2463 (2.74%) | 894 (3.86%) | 1182 (3.90%) | 0.067 | 0.004 |
| Hospitalization | 29030 (20.34%) | 15133 (16.93%) | 5731 (24.83%) | 8166 (27.01%) | 0.252 | 0.003 |
| | | | | | | |
| **Diagnosis (Negatively related-clinically exacerbated)** | | | | | | |
| Syncope | 12979 (9.06%) | 7637 (8.49%) | 2266 (9.79%) | 3076 (10.16%) | 0.058 | 0.002 |
| Chronic seizures/ epilepsy | 6985 (4.87%) | 3963 (4.41%) | 1160 (5.01%) | 1862 (6.15%) | 0.081 | 0.004 |
| Falls | 15145 (10.57%) | 8368 (9.31%) | 2830 (12.23%) | 3947 (13.04%) | 0.121 | 0.002 |
| Fractures | 13302 (9.28%) | 7511 (8.35%) | 2573 (11.12%) | 3218 (10.63%) | 0.095 | 0.005 |
| Pneumonia | 10438 (7.28%) | 4928 (5.48%) | 2254 (9.74%) | 3256 (10.75%) | 0.203 | 0.003 |
| Hyperthyroidism | 6505 (4.54%) | 3804 (4.23%) | 1128 (4.88%) | 1573 (5.20%) | 0.046 | 0.002 |
| Heart failure | 32848 (22.92%) | 15136 (16.84%) | 7009 (30.29%) | 10703 (35.35%) | 0.441 | 0.007 |
| Dyslipidemia | 99984 (69.76%) | 61606 (68.52%) | 16280 (70.36%) | 22098 (72.99%) | 0.097 | 0.012 |
| Narrow angle glaucoma | 22911 (15.99%) | 14665 (26.31%) | 3573 (15.44%) | 4673 (15.44%) | 0.024 | 0.005 |
| Myasthenia gravis | 271 (0.19%) | 152 (0.17%) | 50 (0.22%) | 69 (0.23%) | 0.014 | 0.001 |
| Myocardial infarction | 3228 (2.25%) | 1585 (1.76%) | 690 (2.98%) | 953 (3.15%) | 0.093 | 0.004 |
| Stroke | 18831 (13.14%) | 10763 (11.97%) | 3368 (14.56%) | 4700 (15.52%) | 0.105 | 0.005 |
| Dysrhythmia | 46197 (32.23%) | 23934 (26.62%) | 9286 (40.13%) | 12977 (42.86%) | 0.348 | 0.009 |
| Chronic constipation | 20815 (14.52%) | 11562 (12.86%) | 3796 (16.41%) | 5457 (18.02%) | 0.147 | 0.003 |
| Benign prostatic hyperplasia | 5387 (3.76%) | 3223 (3.58%) | 896 (3.87%) | 1268 (4.19%) | 0.032 | 0.009 |

| | | | | | | |
|---|---|---|---|---|---|---|
| Parkinson' s disease | 9324 (6.51%) | 5364 (5.97%) | 1498 (6.47%) | 2462 (8.13%) | 0.088 | 0.011 |
| Lewy body disease | 6563 (4.58%) | 3900 (4.34%) | 1098 (4.75%) | 1565 (5.17%) | 0.04 | 0.003 |
| | | | | | | |
| **Diagnosis (Positively related-necessitates ACH)** | | | | | | |
| Behavioral and psychological symptoms (BPSD) | 20167 (14.07%) | 10037 (11.16%) | 4338 (18.75%) | 5792 (19.13%) | 0.229 | 0.007 |
| Mood disorders | 17711 (12.36%) | 8542 (9.50%) | 3423 (14.79%) | 5746 (18.98%) | 0.288 | 0.006 |
| Anxiety | 25638 (17.89%) | 12967 (14.42%) | 5043 (21.80%) | 7628 (25.20%) | 0.281 | 0.007 |
| Urinary incontinence | 16608 (11.59%) | 8806 (9.79%) | 2579 (11.15%) | 5223 (17.25%) | 0.233 | 0.007 |
| Muscle spasm/ lower back pain | 52546 (36.66%) | 31133 (34.63%) | 8853 (38.26%) | 12560 (41.49%) | 0.024 | 0.011 |
| Depression | 55451 (38.69%) | 29433 (32.74%) | 10462 (45.22%) | 15556 (51.38%) | 0.383 | 0.01 |
| Abnormal involuntary movements | 34895 (24.35%) | 19471 (21.66%) | 6366 (27.51%) | 9058 (29.92%) | 0.193 | 0.006 |
| Gastrointestinal reflux disease | 36761 (25.65%) | 19879 (22.11%) | 6868 (29.68%) | 10014 (33.08%) | 0.251 | 0.009 |
| Insomnia | 12780 (8.92%) | 6773 (7.53%) | 2480 (10.72%) | 3527 (11.65%) | 0.144 | 0.005 |
| Irritable bowel disease | 3461 (2.41%) | 1877 (2.09%) | 623 (2.69%) | 961 (3.17%) | 0.071 | 0.004 |
| Neuropathic pain | 2394 (1.67%) | 1405 (1.56%) | 388(1.68% ) | 601 (1.99%) | 0.033 | 0.002 |
| Secondary Parkinsonism | 1069 (0.75%) | 592 (0.66%) | 141 (0.61%) | 336 (1.11%) | 0.058 | 0.005 |
| | | | | | | |
| **Claims-based frailty index** | 0.21±0.06 | 0.20±0.06 | 0.22±0.06 | 0.23±0.07 | | |
| Robust | 28962 (20.21%) | 21677 (24.11%) | 3376 (14.59%) | 3909 (12.91%) | | |
| Prefrail | 71097 (49.61%) | 46611 (51.84%) | 10829 (46.80%) | 13657 (45.11%) | | |
| Mild frail | 39491 (27.55%) | 20261 (22.54%) | 7998 (34.57%) | 11232 (37.10%) | | |
| Moderate to severe frail | 3770 (2.63%) | 1358 (1.51%) | 935 (4.04%) | 1477 (4.88%) | 0.19 | 0.006 |
| | | | | | | |

**Supplementary Table S9. Extent and time to censoring across study groups.**

| Stratum | Extent of censoring (%) | Mean Time (time in months) | Standard Error |
|---|---|---|---|
| **No/Low burden** | 27,339 (30.41) | 8.8381 | 0.0172 |
| **Moderate burden** | 7,894 (34.12) | 6.9572 | 0.0445 |
| **High burden** | 10,943 (36.15) | 7.4409 | 0.0346 |

**FIGURES**

**Supplementary Figure S1. Overlap of propensity scores in each exposure group.**

A) Low-no burden

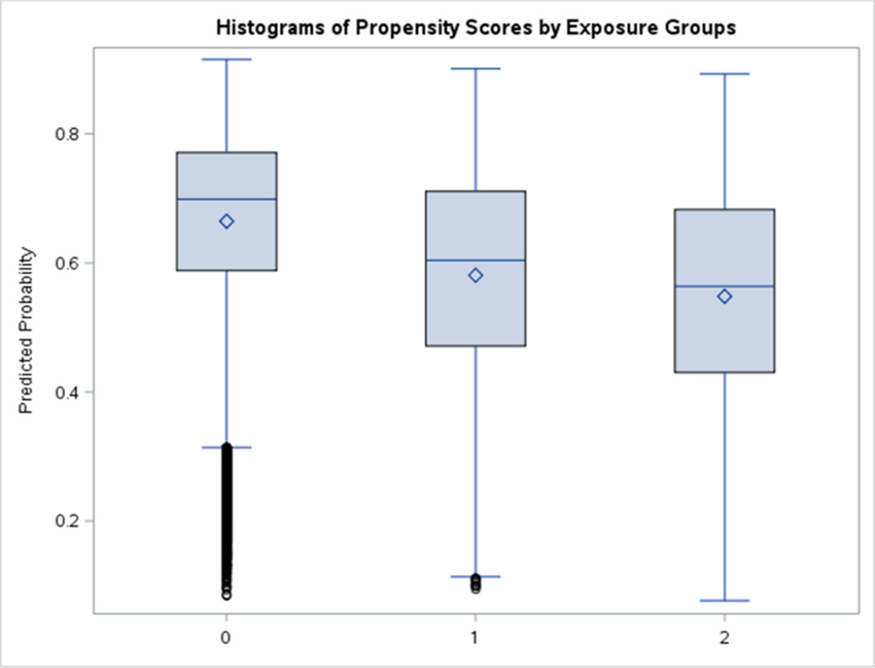


B) Moderate burden

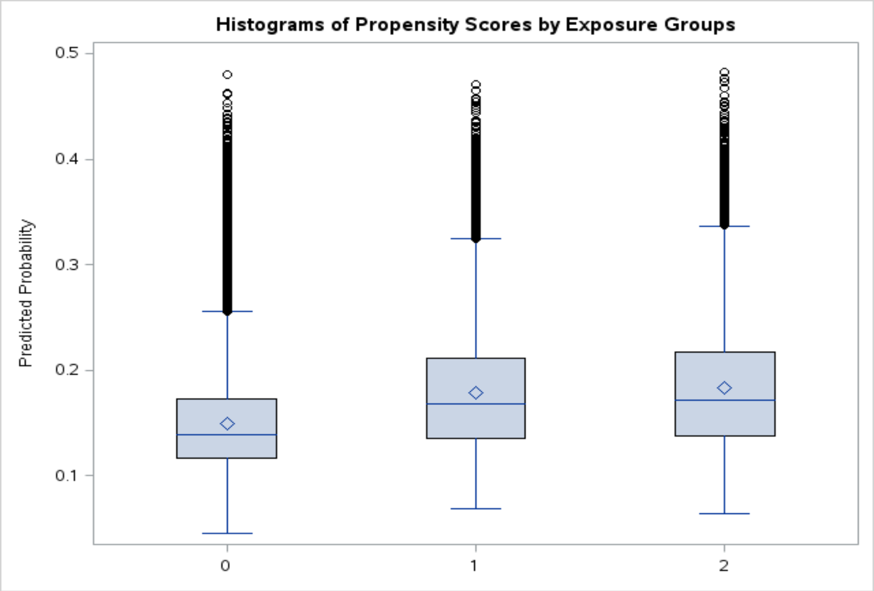


C) High burden

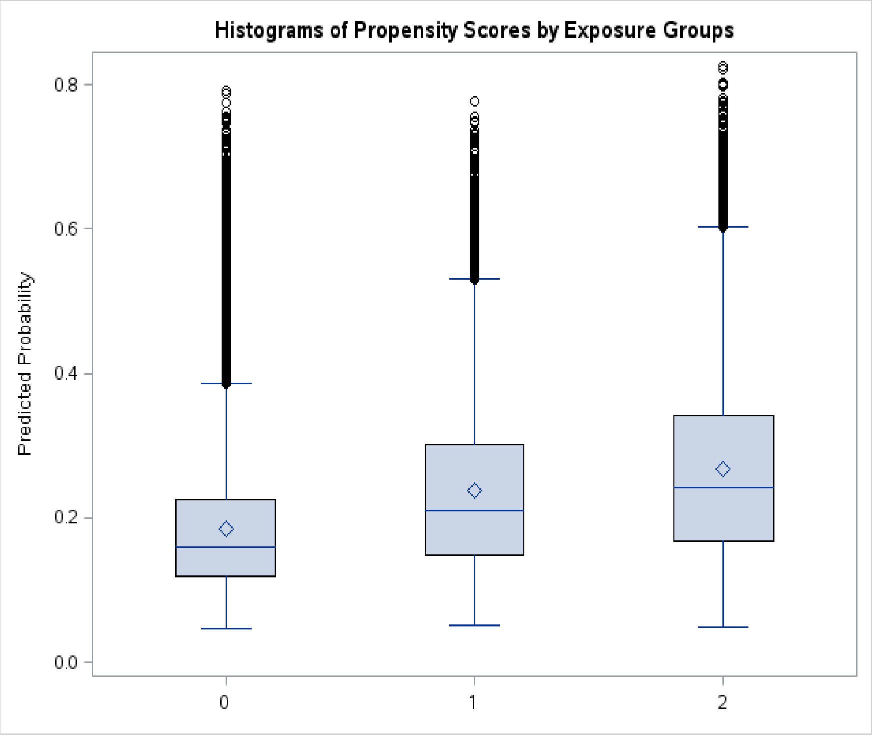
Histograms of Propensity Scores by Exposure Groups
Predicted Probability
0.8
0.6
0.4
0.2
0.0
0
1
2

**Supplementary Figure S2. Balancing using generalized boosting method for multiple propensity scores.**

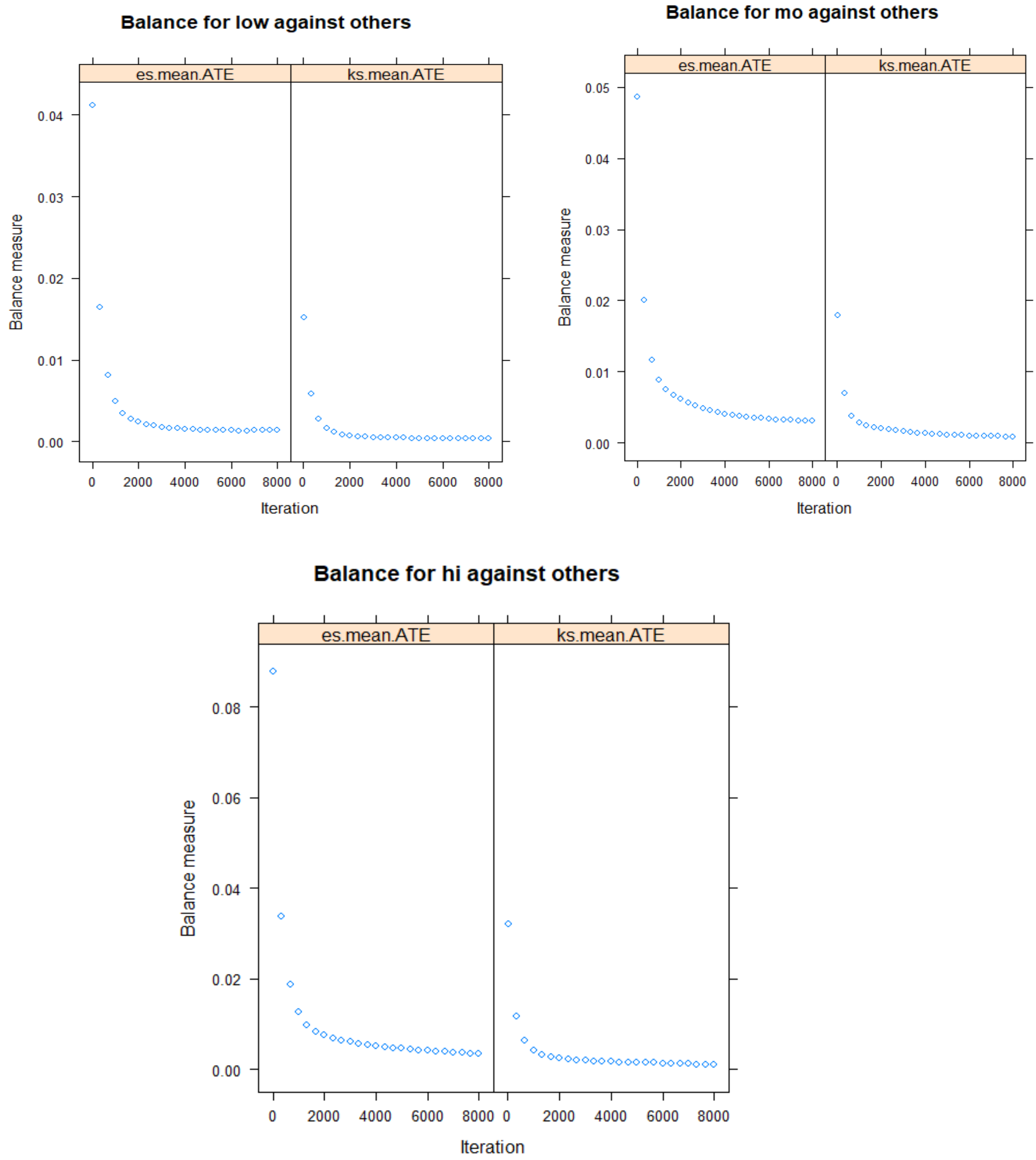

**Supplementary Figure S3. Overlap of propensity scores calculated for multiple propensity score weighting using generalized boosting methods.**

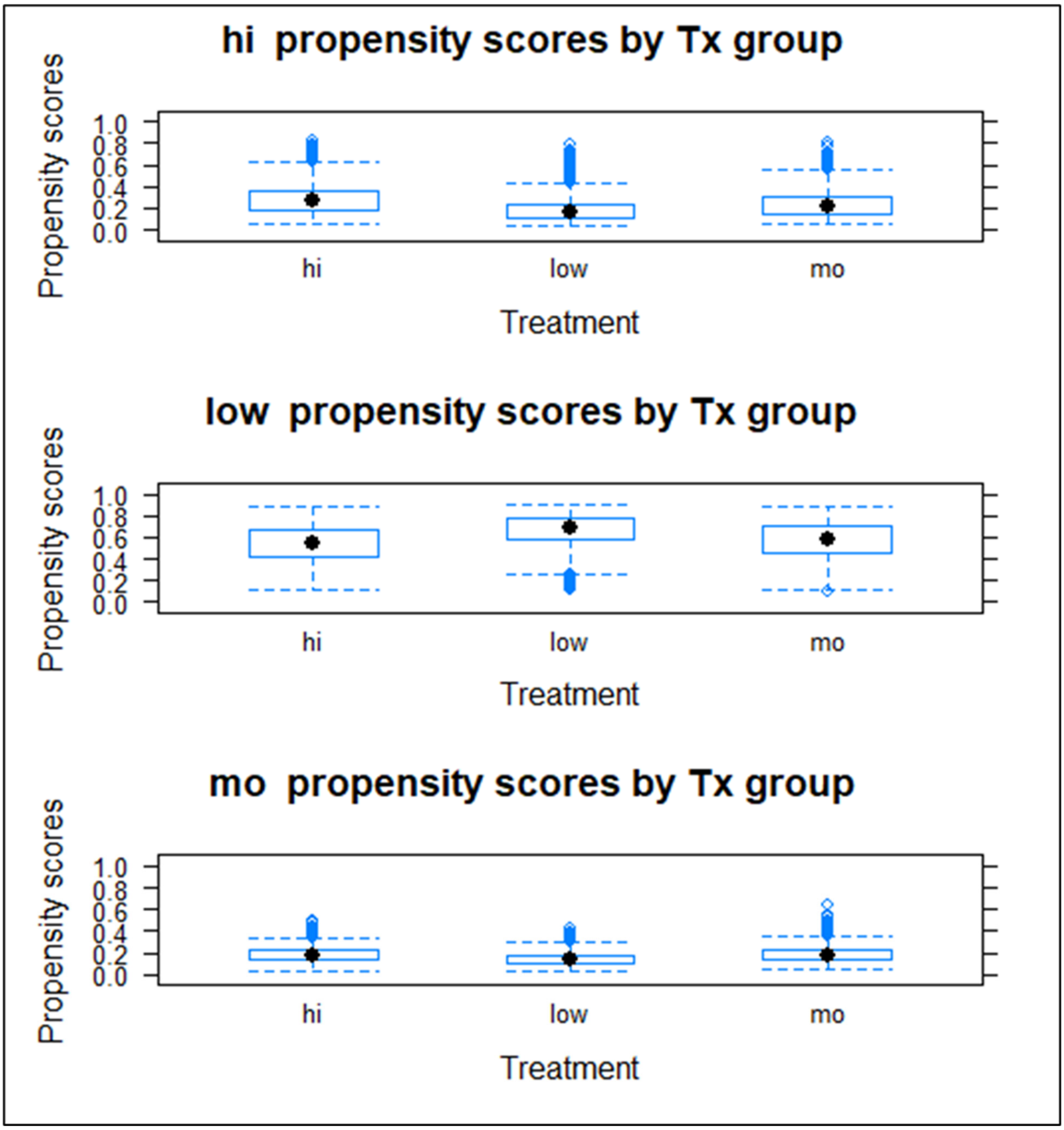

**Supplementary Figure S4. Log negative Log plot**

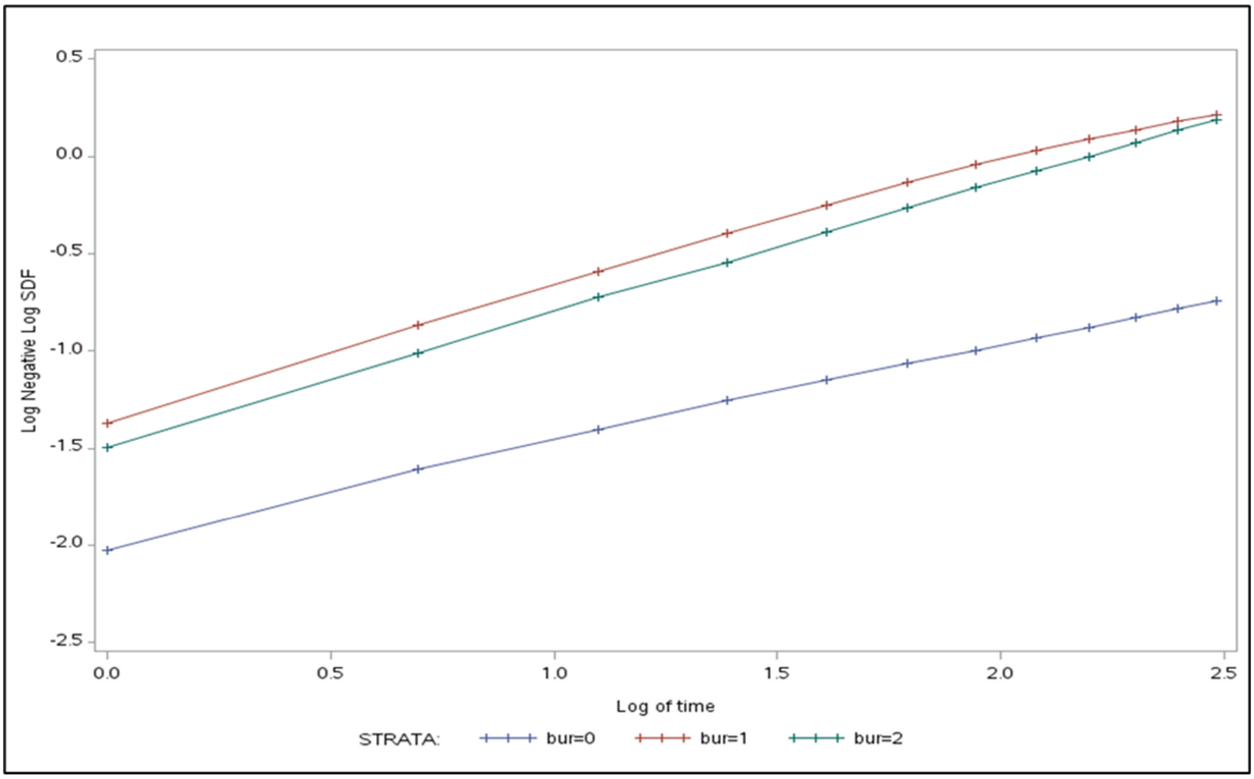

## STROBE Checklist [12]for *cohort studies.*

| | Item No. | Recommendation | Page No. |
|---|---|---|---|
| **Title and abstract** | 1 | (*a*) Indicate the study's design with a commonly used term in the title or the abstract | 3 |
| | | (*b*) Provide in the abstract an informative and balanced summary of what was done and what was found | 3 |
| **Introduction** | | | |
| Background/rationale | 2 | Explain the scientific background and rationale for the investigation being reported | 4,5 |
| Objectives | 3 | State specific objectives, including any prespecified hypotheses | 5 |
| **Methods** | | | |
| Study design | 4 | Present key elements of study design early in the paper | 6 |
| Setting | 5 | Describe the setting, locations, and relevant dates, including periods of recruitment, exposure, follow-up, and data collection | 5,6 |
| Participants | 6 | (*a*) Give the eligibility criteria, and the sources and methods of selection of participants. Describe methods of follow-up | 7 |
| | | (*b*) For matched studies, give matching criteria and number of exposed and unexposed | - |
| Variables | 7 | Clearly define all outcomes, exposures, predictors, potential confounders, and effect modifiers. Give diagnostic criteria, if applicable | 7 |
| Data sources/ measurement | 8 | For each variable of interest, give sources of data and details of methods of assessment (measurement). Describe comparability of assessment methods if there is more than one group | *5* |
| Bias | 9 | Describe any efforts to address potential sources of bias | 9 |
| Study size | 10 | Explain how the study size was arrived at | 7 |
| Quantitative variables | 11 | Explain how quantitative variables were handled in the analyses. If applicable, describe which groupings were chosen and why | 8 |
| Statistical methods | 12 | (*a*) Describe all statistical methods, including those used to control for confounding | 9 |
| | | (*b*) Describe any methods used to examine subgroups and interactions | 10 |
| | | (*c*) Explain how missing data were addressed | 6 |
| | | (*d*) If applicable, explain how loss to follow-up was addressed | 6 |
| | | (*e*) Describe any sensitivity analyses | 10 |
| **Results** | | | |
| Participants | 13 | (a) Report numbers of individuals at each stage of study—e.g. numbers potentially eligible, examined for eligibility, confirmed eligible, included in the study, completing follow-up, and analyzed | 23 |
| | | (b) Give reasons for non-participation at each stage | 23 |
| | | (c) Consider use of a flow diagram | 23 |

| | | | |
|---|---|---|---|
| Descriptive data | 14 | (a) Give characteristics of study participants (e.g. demographic, clinical, social) and information on exposures and potential confounders | 24 |
| | | (b) Indicate number of participants with missing data for each variable of interest | 11 |
| | | (c) Summarize follow-up time (e.g., average and total amount) | 11 |
| Outcome data | 15 | Report numbers of outcome events or summary measures over time | 26,27 |
| Main results | 16 | (*a*) Give unadjusted estimates and, if applicable, confounder-adjusted estimates and their precision (e.g., 95% confidence interval). Make clear which confounders were adjusted for and why they were included | 11 |
| | | (*b*) Report category boundaries when continuous variables were categorized | 26,27 |
| | | (*c*) If relevant, consider translating estimates of relative risk into absolute risk for a meaningful time period | - |
| Other analyses | 17 | Report other analyses done—e.g. analyses of subgroups and interactions, and sensitivity analyses | 12 |
| **Discussion** | | | |
| Key results | 18 | Summarize key results with reference to study objectives | 12 |
| Limitations | 19 | Discuss limitations of the study, taking into account sources of potential bias or imprecision. Discuss both direction and magnitude of any potential bias | 14 |
| Interpretation | 20 | Give a cautious overall interpretation of results considering objectives, limitations, multiplicity of analyses, results from similar studies, and other relevant evidence | 13,14 |
| Generalizability | 21 | Discuss the generalizability (external validity) of the study results | 13-15 |
| **Other information** | | | |
| Funding | 22 | Give the source of funding and the role of the funders for the present study and, if applicable, for the original study on which the present article is based | 16 |

# REFERENCES


1. Campbell, N., et al., *The 2012 update to the anticholinergic cognitive burden scale.* Journal of the American Geriatrics Society, 2013. **61**(S1): p. S142-S143.
2. Tayebati, S.K., et al., *Identification of World Health Organisation ship's medicine chest contents by Anatomical Therapeutic Chemical (ATC) classification codes.* International maritime health, 2017. **68**(1): p. 39-45.
3. *WHO Collaborating Centre for Drug Statistics Methodology. Guidelines for ATC classification and DDD assignment 2020. Oslo: WHO Collaborating Centre for Drug Statistics Methodology;2019.*
4. Israni, J., et al., *Delirium as a predictor of mortality in US Medicare beneficiaries discharged from the emergency department: a national claims-level analysis up to 12 months.* BMJ Open, 2018. **8**(5): p. e021258.
5. Chuen, V.L., et al., *Assessing the Accuracy of International Classification of Diseases (ICD) Coding for Delirium.* J Appl Gerontol, 2022. **41**(5): p. 1485-1490.
6. Bell, J.S., et al., *Sedative load among long-term care facility residents with and without dementia: a cross-sectional study.* Clin Drug Investig, 2010. **30**(1): p. 63-70.

7. Linjakumpu, T., et al., *A model to classify the sedative load of drugs.* Int J Geriatr Psychiatry, 2003. **18**(6): p. 542-4.
8. Ah, Y.-M., et al., *Change in sedative burden after dementia onset using difference-in-difference estimations.* PloS one, 2019. **14**(8): p. e0220582.
9. *U.S Department of Health & Human Services Guidance Portal.Opioid Oral Morphine Milligram Equivalent (MME) Conversion Factors table for prescription drug coverage www.hhs.gov/guidance/document/opioid-oral-morphine-milligram-equivalent-mme-conversion-factors-0. (Accessed on: Feb 09, 2023)*.
10. *Centers for Medicare & Medicaid Services. Opioid Morphine Equivalent Conversion Factors. 2017;1. https://medicaid.utah.gov/Documents/files/Opioid-Morphine-EQ-Conversion-Factors.pdf (Accessed on: Feb 09, 2023)*.
11. *Centers of Medicare & Medicaid Services. Calculating total daily dose of opioids for safer dosagehttps://www.cdc.gov/opioids/providers/prescribing/pdf/calculating-total-daily-dose.pdf (Accessed on: Feb 09, 2023)*.
12. von Elm, E., et al., *The Strengthening the Reporting of Observational Studies in Epidemiology (STROBE) Statement: guidelines for reporting observational studies.* Int J Surg, 2014. **12**(12): p. 1495-9.
